# Beneficial Effects of Pomegranate Peel Extract and Probiotics on Pre-adipocyte Differentiation

**DOI:** 10.3389/fmicb.2019.00660

**Published:** 2019-04-03

**Authors:** Valeria Sorrenti, Cinzia Lucia Randazzo, Cinzia Caggia, Gabriele Ballistreri, Flora Valeria Romeo, Simona Fabroni, Nicolina Timpanaro, Marco Raffaele, Luca Vanella

**Affiliations:** ^1^Dipartimento di Scienze del Farmaco, Sezione di Biochimica, Università di Catania, Catania, Italy; ^2^Dipartimento di Agricoltura, Alimentazione e Ambiente – Di3A, Università di Catania, Catania, Italy; ^3^Council for Agricultural Research and Economics (CREA) – Research Centre for Olive, Citrus and Tree Fruit, Acireale, Italy

**Keywords:** lactobacilli, antimicrobial activity, pomegranate extract, adipocyte differentiation, combining foods

## Abstract

The beneficial effects of pomegranate are due to the ellagitannins and anthocyanins content, which are protective toward a wide variety of diseases including inflammatory diseases. Many investigators have reported that pomegranate waste (peel and seeds) extracts, made from waste product of industrial processing, show free radical scavenger and a potent antioxidant capacity. Pomegranate extracts (PEs) were also reported to possess noteworty antibacterial, antiviral, hypolipidemic, and anti-inflammatory bioactivities thanks to the polyphenolic compounds content, which includes punicalagins, gallic acid, and ellagic acid derivatives. The focus of the present manuscript was to study the prebiotic potentiality of a PE, soluble in water, and characterized through HPLC–PDA–ESI/MS*^n^* for its phenolic content. Moreover, since it has been reported that pomegranate extracts decreased the level of lipids in the blood and that a number of probiotic strains have been shown to affect adipogenesis in cell culture, this study was also performed to test the *in vitro* effects of PE and probiotic *L. rhamnosus* GG ATCC 53103 strain (LGG) on 3T3-L1 cell line. PE and probiotics substantially reduced the triglyceride content and intracellular lipid increase, compared to the control group. However, the combination treatment of PE and LGG filtered spent broth (SB) was the most effective in reducing triglyceride content and intracellular lipid accumulation. The mRNA expression levels of the main transcriptional factors implicated in adipocyte differentiation were substantially lower in 3T3-L1 cells treated with PE and LGG filtered SB. These results evidenced that a synergistic effect of probiotics and polyphenols contained in PE may affect *in vitro* adipogenesis and may contribute in development of new nutraceutical/probiotic-based remedies to prevent and to treat obesity.

## Introduction

Pomegranate is a fruit whose positive health effects have been extensively studied. This fruit is rich in bioactive compounds such as ellagitannins and anthocyanins content, which are protective toward degenerative diseases. Pomegranate fruit, because of its high nutritive value, health benefits, and antioxidant bioactive compounds, is considered as a food medicine. In fact, pomegranate has been considerably used in herbal medicine for several pathologies including flu and infections of the upper respiratory tract. All parts of the pomegranate fruit, i.e., peel and seeds, considered as waste products, can be processed for value-added products having industrial, medicinal, and cosmetic value (Dhumal et al., 2014).

Pomegranate wastes are produced in all the phases of fruits life cycle, i.e., during agricultural production, industrial manufacturing, and processing. It is possible to take advantage of pomegranate by-products as they are a rich source of bioactive compounds such as flavonoids, phenolic acids, and tannins. Moreover, many researchers have described that pomegranate extracts, made from by-products of the processing factories, have an effective free radical scavenging activity and antioxidant capacity (Lee et al., 2010; Panichayupakaranant et al., 2010; Fischer et al., 2011).

Furthermore, the pomegranate extracts act as natural inhibitors of pathogens, bacteria, and fungi (Al-Zoreky, 2009; Tehranifar et al., 2011; Romeo et al., 2015).

Pomegranate ellagitannins are hydrolyzed by gut microbiota to smaller phenolics, such as ellagic acid. Ellagic acid is then absorbed into the blood circulation, while ellagitannins are not absorbed and are metabolized into urolithins.

It has been reported that pomegranate by-products and punicalagins significantly are able both to inhibit the growth of pathogenic *Escherichia coli*, *Pseudomonas aeruginosa*, *Clostridia*, and *Staphylococcus aureus* (Reddy et al., 2007; Bialonska et al., 2009) and to increase the growth on beneficial bacteria including *Bifidobacterium* spp. and *Lactobacillus* spp. (Reddy et al., 2007; Bialonska et al., 2010).

Pomegranate extracts were also reported to decrease the level of lipids in the blood and to have significant anticancer, antiviral, and anti-inflammatory activities (Li et al., 2006; Hossin, 2009; Lin et al., 2013; Bassiri-Jahromi, 2018).

These potential beneficial effects are attributed to the polyphenolic compounds that the pomegranate extracts contain including punicalagins, gallic acid, and ellagic acid derivatives (Vanella et al., 2013a,b,c; Romeo et al., 2015).

Because obesity is one of the main public health problems, new preventive strategies are necessary (Smith and Smith, 2016).

Adipocyte plays a major role in the begin or development of metabolic complications associated to obesity, such as metabolic syndrome and diabetic complications (Kim and Plutzky, 2016).

The interest of the researchers in the identification of natural products obtained from dietary plants that have anti-obesity activities has increased. It has been reported that the xanthigen and fucoxanthin, natural compounds of pomegranate seed oil, significantly suppressed adipocyte differentiation and lipid accumulation (Lai et al., 2012).

The focus of the present manuscript was firstly to study the antioxidant and antimicrobial activities, and the prebiotic potential of a PE rich in phenolic compounds. Moreover, the enriched standardized PE, containing high percentages of pomegranate natural antioxidants, was chemically characterized through HPLC–PDA–ESI/MSn. Secondly, since pomegranate juice (Les et al., 2018) and different types of pomegranate extracts (PEs), including extract prepared from the whole fruit (Li et al., 2015) and extract derived from pomegranate peel (Neyrinck et al., 2013), and a number of probiotic strains (Moon et al., 2012; Park et al., 2014) have been shown to affect adipogenesis, this study was carried out to test the *in vitro* effects of PE, probiotic *L. rhamnosus* GG ATCC 53103 (LGG) preincubated with PE, alone or in combination, on 3T3-L1 cell differentiation.

## Materials and Methods

### Chemicals

The powdered pomegranate extract (Dermogranate^®^) employed in this study was provided by Medinutrex (Catania, Italy). Briefly, the extract was prepared from dried and grinded pomegranate fruits mixed with hydroalcoholic solutions (food grade) and then filtered. The filtrate was concentrated and then spray-dryed to obtain the standardized extract. The Dermogranate^®^ extract had the following chemical composition: total polyphenols (16%), punicalagins (8%), ellagic acid, and derivatives (8%).

Folin–Ciocalteu reagent (FCR), sodium carbonate (Na_2_CO_3_), gallic acid, punicalin (mixture of anomers), punicalagin, and ellagic acid were purchased from Sigma-Aldrich (Milan, Italy). Granatin B was purchased from LGC Standards (London, United Kingdom). HPLC–MS grade solvents (Merck KgaA, Darmstadt,Germany) were used for chromatography and all other reagents were of analytical grade.

### Determination of Total Polyphenols Content

The Folin–Ciocalteu assay (Singleton et al., 1999) was used for the determination of total polyphenols content with slight modifications. 0.1 mg/ml of extract was dissolved in distilled water. Then, 5 ml of 10% FCR and 4.5 ml of Na_2_CO_3_ solution (7.5% w/v) were added to 500 μl of sample. The final solution was agitated for 2 h in the dark and then the Abs at λ = 765 nm was measured. Analyses were carried out in triplicate and the concentration of total polyphenols was expressed as g of gallic acid equivalents (GAEs)/100 g of extract.

### HPLC–PDA–ESI/MS*^n^* Analysis of Phenolic Compounds

Separation and quantification of phenolic compounds were performed as previously described (Romeo et al., 2015). For the identification of phenolic compounds, the retention times (RTs), spectra, and MS data in negative ESI mode were compared to those of authentic standards. Quantification of each phenolic compound was performed using the corresponding standard as external standard. Quantification was carried out at 280 nm for gallic acid. Punicalins, granatin B, punicalagins, and ellagic acid were quantified at 378 nm; the same wavelength was used for the quantification of ellagic acid derivatives using ellagic acid as reference standard. Analyses were carried out in triplicate and the results were expressed as g of compound/100 g of extract.

### Quenching of DPPH

The free radical-scavenging capacity of different concentrations of PE extract (3.4–1.7–0.85–0.56–0.42–0.34–0.21–0.17–0.11–0.085–0.028 mg/ml) was measured by 2,2-diphenyl-1-picryl-hydrazyl-hydrate (DPPH)-free radical method as previously reported (Salerno et al., 2012). Results are expressed as percentage of inhibition rate ±*SD*.

### Scavenger Effect on Superoxide Anion

The superoxide anion-scavenging capacity of different concentrations of PE extract (3.4–1.7–0.85–0.56–0.42–0.34–0.21–0.17–0.11–0.085–0.028 mg/ml) was measured as previously reported (Salerno et al., 2012). Results are expressed as percentage of inhibition rate ± SD.

### 3T3-L1 Murine Pre-adipocytes Cell Viability

3T3-L1 murine pre-adipocytes were bought from American Type Culture Collection (Rockville, MD, United States). Cells were plated at a concentration of 2 × 10^5^ cells per well of a 96-well microplate and cultured at 37°C in incubator with 5% CO_2_ for 48 h in the absence and presence of the different concentrations of PE reported above. Cell viability was measured by MTT assay as previously reported (Di Giacomo et al., 2015). MTT, a yellow tetrazole, is reduced to purple formazan in living cells. Results are expressed as percentage of formazan produced in treated 3T3-L1 murine pre-adipocytes cells compared to untreated cells.

### Microbiology

#### Bacterial Cultivation

The commercial pathogen strains *E. coli* ATCC 25922, *S. aureus* ATCC 29213, *Listeria innocua* ATCC 33090, and *Salmonella enterica* ATCC 14028 were used. *E. coli* strain was grown in Luria-Bertani (LB) broth at 37°C overnight; *S. aureus*, *S. enterica*, and *L. innocua* were routinely grown overnight, in Tryptone Soya Broth (TSB) at 37 and 30°C, respectively. All media and supplements were provided by Oxoid (Milan, Italy).

The commercial probiotic strains LGG, *Bifidobacterium animalis* BB12, *B. longum* BB536, and the wild strain *Lactobacillus paracasei* N 24, isolated from Pecorino crotonese cheese, were cultured in deMan–Rogosa–Sharpe (MRS) broth at 37°C overnight.

Overnight bacterial culture was incubated at 37°C for 24 h, under anaerobic condition, until they reached a cell density of approximately 1.0 × 10^9^ cfu/ml.

#### Growth Rate Determination

Based on results obtained on free radical scavenger activity of PE and on 3T3-L1 murine pre-adipocytes cell viability experiments, different concentrations were used for treatment of probiotic or pathogen strains as described below.

Antimicrobial activity of the PE was evaluated against the commercial pathogen strains mentioned above. Overnight pathogen cultures were co-cultured at 37°C for 24 h with PE at different concentrations (1.7–0.34–0.17 mg/ml), and the antimicrobial activity was evaluated by plating count of live bacteria and expressed as cfu/ml.

The effect of PE on the growth of probiotic strains mentioned above was evaluated inoculating in co-culture the probiotic strains at a cell density of approximately 1.0 × 10^9^ cfu/ml with the PE at different concentrations (0.085–0.042–0.028 mg/ml). The effect of the extract on growth of probiotic strains was evaluated after incubation at 37°C for 24 h under anaerobic conditions by plating count of live bacteria and expressed as cfu/ml.

All experiment was conducted in duplicate and results were expressed as mean values and standard deviation. Based on preliminary results LGG was chosen for the subsequent analyses.

Fresh broth cultures were centrifuged at 5000 rpm for 10 min at 4°C, and the supernatant was decanted to collect the spent broth (SB), which was filtered (FSB), using a 0.22-μm filter and then used for further analyses.

The bacterial pellet was resuspended in 1 ml of PBS and sonicated five times at 44% amplitude for 2 min with 6 min of rest. The sonicate was then centrifuged at 1100 ×*g* for 15 min at 4°C. The supernatant was collected, filtered through a 0.22-μm filter, and labeled bacterial cell extract (CE). The CE was used for further analysis.

### Cell Culture and Adipocyte Cell Differentiation

3T3-L1 murine pre-adipocytes were resuspended in Dulbecco’s Modified Eagle Medium (DMEM), containing 10% fetal bovine serum (FBS, Invitrogen, Carlsbad, CA, United States) and 1% antibiotic/antimycotic solution (Invitrogen, Carlsbad, CA, United States) and seeded in a 75-cm^2^ flask at a density of 1 to 2 × 10^4^ cells. Adipocyte cell differentiation was obtained as previously reported (Waldman et al., 2016).

Differentiating 3T3-L1 pre-adipocytes were treated for 7 days with PE (0.028 mg/ml), LGG CE (25 μg/ml), and LGG filtered SB (10 μg/ml) from overnight bacterial culture incubated with or without PE (0.028 mg/ml).

### Lipid Content Quantification

To quantify lipid accumulation, Oil Red Staining was performed as previously reported (Barbagallo et al., 2017). Formation of lipid drops was measured with an inverted multichannel LED fluorescence microscope (Evos, Life Technologies, Grand Island, NY, United States).

### RNA Extraction and qRT-PCR

Expressions of adiponectin, PPAR-γ, SREBP, FAS, IL-6, and IL-10 were evaluated by real-time PCR. RNA was extracted and quantified as previously reported (Raffaele et al., 2018). Appropriate primer sequences were used (Table 1). The relative mRNA expression level was measured by the threshold cycle (Ct) value of each PCR product and normalized with that of GAPDH by using comparative 2^-ΔΔCt^ method.

**Table 1:** PCR primers used in this study.

Gene	Forward primer	Reverse primer
Adiponectin	GAAGCCGCTTATGTGTATCGC	GAATGGGTACATTGGGAACAGT
IL-6	TTCCTCTCTGCAAGAGACTTCC	AGGAGAGCATTGGAAATTGGGG
FAS	GGAGGTGGTGATAGCCGGTAT	TGGGTAATCCATAGAGCCCAG
GAPDH	AGCTTCGGCACATATTTCATCTG	CGTTCACTCCCATGACAAACA
IL-10	GCTGGACAACATACTGCTAACC	ATTTCCGATAAGGCTTGGCAA
SREBP-1	GATGTGCGAACTGGACACAG	CATAGGGGGCGTCAAACAG
PPAR-γ	TCGCTGATGCACTGCCTATG	ACCTGATGGCATTGTGAGACAT


### Statistical Analyses

Statistical analyses of multiple comparisons were performed by the Fisher method. *P*-values lower than 0.05 were accepted as significant. Data were analyzed using either single-factor analysis of variance (ANOVA) for multiple groups, or the unpaired *t*-test for two groups, and the results are presented as mean ± SD.

## Results

### HPLC–PDA–ESI/MS*^n^* Analysis of PE

**FIGURE 1:**
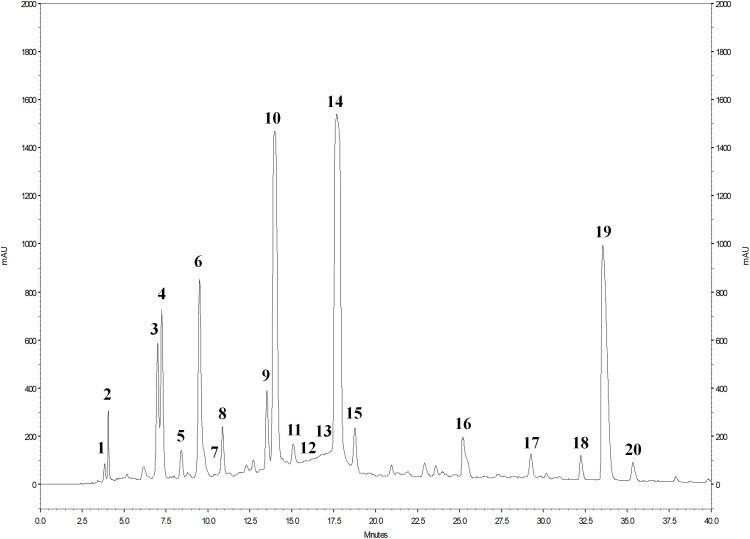
HPLC chromatogram of phenolic compounds of PE detected at 378 nm. For the identification of the peaks, see Table 2.

The phenolic profile of PE (Figure 1) included the determination of 1 hydroxybenzoic acid and 19 ellagitannins. The main peaks corresponded to punicalin (peak 4), granatin B (peak 6), punicalagin A and B (peaks 10 and 14), and ellagic acid (peak 19) (Figure 1). The presence of gallic acid (peak 1) and ellagic acid derivatives (peaks 2, 3, 5, 7–9, 11–13, 15–18, and 20) was also revealed. As shown in the chromatogram, ellagitannins are the predominant class of phenolic compounds in pomegranate peel and marc (a by-product made up of seeds and peels), since they represent over the 99% of the total content of pomegranate phenolics. Punicalagins, the major ellagitannins of pomegranate by-products, accounted for 47.6% of the total phenolics content in PE (Table 2). Ellagic acid derivatives, ellagic acid, and other minor phenolic compounds (punicalin, granatin B, and gallic acid), accounted for 38.4, 10.2, and 3.8% of the total phenolics content in PE, respectively (Table 2).

**Table 2:** Peak list and quantification of the phenolics in PE.

Peak number^a^	RT (min)	λ (nm)	[M–H]^-^ (*m*/*z*)	MS*^n^* (*m*/*z*)	Phenolic compounds	g/100 g^b^
1	3.9	269,310	169	125	Gallic acid	0.07 ± 0.02
2	4.1	255,364	–	301	Ead^d^	0.03 ± 0.01
3	7.0	255,363	–	301	Ead^d^	0.41 ± 0.04
4	7.3	263,364	781	601	Punicalin	0.27 ± 0.04
5	8.4	264,366	–	301	Ead^d^	0.19 ± 0.09
6	9.5	260,365	951	933/613	Granatin B	0.28 ± 0.03
7	10.4	259,361	–	301	Ead^d^	0.02 ± 0.01
8	10.9	257,360	–	301	Ead^d^	0.05 ± 0.01
9	13.5	256,362	–	301	Ead^d^	1.23 ± 0.04
10	14.0	258,378	1083	781/601	Punicalagin A	3.05 ± 0.05
11	15.1	257,363	–	301	Ead^d^	0.13 ± 0.09
12	15.9	257,361	–	301	Ead^d^	0.95 ± 0.03
13	16.8	258,360	–	301	Ead^d^	2.69 ± 0.11
14	17.7	257,378	1083	781/601	Punicalagin B	4.77 ± 0.21
15	18.8	257,362	–	301	Ead^d^	0.12 ± 0.01
16	25.2	256,363	–	301	Ead^d^	0.17 ± 0.09
17	29.3	254,361	–	301	Ead^d^	0.17 ± 0.01
18	32.2	255,360	–	301	Ead^d^	0.08 ± 0.03
19	33.6	256,367	301	229/185	Ellagic acid	1.68 ± 0.01
20	35.4	248,362	–	301	Ead^d^	0.08 ± 0.02
Total polyphenols^c^						16.48 ± 2.49
Punicalagins						7.82
Ellagic acid derivatives						6.31
Ellagic acid						1.68
Other phenolic compounds						0.62
Total						16.43


*^a^The numbering is according to Figure 1. ^*b*^Results are expressed as the mean ± standard deviation. ^*c*^Expressed as g GAE/100 g. ^*d*^Ellagic acid derivative (Ead).*

The mass spectrometric properties of the 20 phenolic compounds identified (peaks 1–20) are shown in Table 2. As previously reported (Romeo et al., 2015), two isomeric forms (A and B) of punicalagins (peaks 10 and 14) were observed, as well as the presence of granatin B (peak 6) was highlighted. Furthermore, these compounds were also characterized by direct infusion-negative ion ESI/MS*^n^* analysis of standard compounds. Peak 4 was identified as punicalin (*m/z* 781) while peaks 2, 3, 5, 7–9, 11–13, 15–18, and 20 were identified as ellagic acid derivatives according to their UV–Vis and mass characteristics (λ_max_ around 370 nm and MS^1^ fragment at *m*/*z* 301 corresponding to ellagic acid).

### Free Radical Scavenging Activity of PE

Antioxidant activity of PE was tested by their ability to reduce the stable DPPH radical.

**FIGURE 2:**
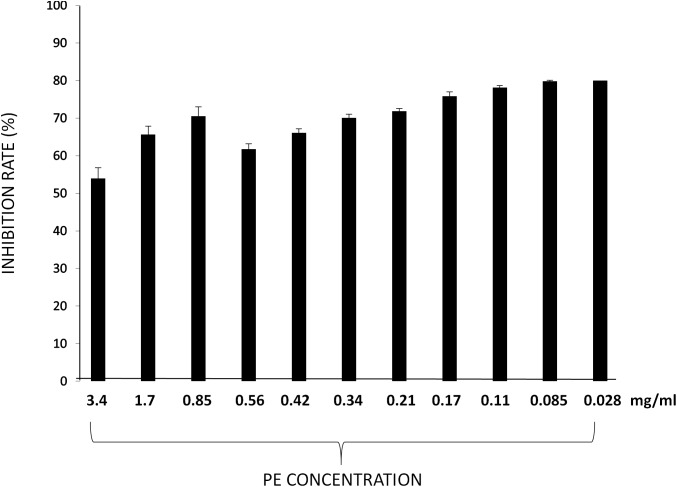
DPPH radical scavenging activities of PE at different concentrations. Results are expressed as percentage of inhibition rate ± SD.

Particularly, the percentage of inhibition of DPPH resulted up to 75% at concentrations lower to 0.21 mg/ml (76,78, 79, and 80% respectively, at concentration of PE of 0.17- 0.11- 0.085-and 0.028 mg/ml). At concentrations higher to 0.21 mg/ml the percentage of inhibition of DPPH resulted lower (Figure 2).

Pomegranate extract inhibited superoxide anion formation in a dose-dependent manner (Table 3). As a general trend, in this test PE resulted more effective than in the previous one. This might be due to the smaller size of superoxide anion compared to DPPH radical.

**Table 3:** Superoxide ion scavenging activities of different concentrations of PE.

PE concentrations	% of inhibition	PE concentrations	% of inhibition
PE (3.4 mg/ml)	65 ± 2	PE (0.21 mg/ml)	78 ± 2
PE (1.7 mg/ml)	73 ± 3	PE (0.17 mg/ml)	80 ± 1
PE (0.85 mg/ml)	75 ± 1	PE (0.11 mg/ml)	85 ± 3
PE (0.56 mg/ml)	70 ± 2	PE (0.085 mg/ml	88 ± 4
PE (0.42 mg/ml)	68 ± 1	PE (0.028 mg/ml)	95 ± 1
PE (0.34 mg/ml)	75 ± 4		


*Results are expressed as percentage of inhibition rate + SD.*

### Effect of PE on 3T3-L1 Cell Viability

**FIGURE 3:**
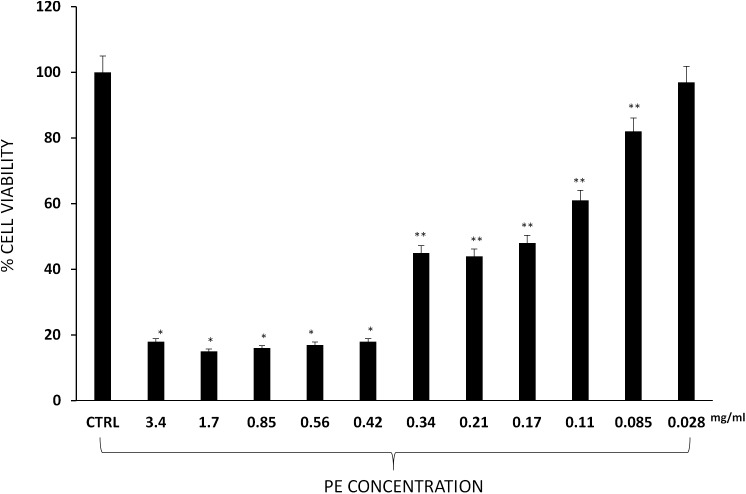
Percentage of 3T3-L1 murine pre-adipocytes survival in the presence of PE at different concentrations. Results are expressed as the means ± SD of four experiments performed in triplicate. Significant vs. untreated controls: ^∗^*p* < 0.005; ^∗∗^*p* < 0.05.

3-(4,5-Dimethylthiazol-2-yl)-2,5-Diphenyltetrazolium Bromide (MTT) assay was carried out to evaluate 3T3-L1 cell viability. Results show a significant reduction of cell viability with high concentrations of PE (3.4, 1.7, 0.85, 0.56, and 0.42 mg/ml), whereas lower (0.34, 0.21, 0.17, 0.11, and 0.085 mg/ml) concentrations had a moderate inhibitory effect and 0.028 mg/ml concentration had no significant effect on 3T3-L1 murine pre-adipocytes cell viability (Figure 3).

### Microbiology

#### Antimicrobial Activity of the PE on Pathogen Strains

**FIGURE 4:**
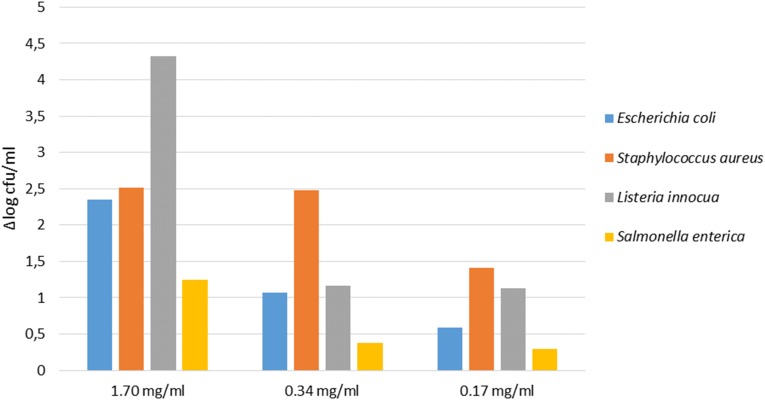
Antimicrobial activity of different concentration of PE on pathogens detected at T24 (24 h of inoculum). The cell density is expressed as Δlog cfu/ml.

Data of co-culture assay have shown that pathogens were differently sensitive to the PE. In detail, as showed in Figure 4, the extract at the concentration of 1.7 mg/ml showed the highest antimicrobial activity against all pathogens, with a significant decrease of *L. innocua* (proximally 4 log unit). At the concentration of 0.34 mg/ml the extract was efficacy against *E. coli*, *L. innocua*, and *S. aureus*, exhibiting a reduction of cell density of 1 and 2 log units, respectively. When the PE was tested at 0.17 mg/ml concentration, *S. aureus*, *S. enterica*, and *L. innocua* strains were still inhibited, while the growth of *E. coli* was not significantly affected.

#### Effect of the PE on Growth of Probiotic Strains

Results shown in Table 4 highlighted that PE, at all tested concentrations, did not have any inhibitory activity on growth of probiotic strains tested. A slight increase in growth was observed for LGG co-cultured with PE at concentration of 0.028 mg/ml (Table 4).

**Table 4:** Bacterial counts expressed as log_10_ cfu/ml of three replicates ± SD of *L. rhamnosus* GG ATCC 53103, *Bifidobacterium animalis* BB12, *B. longum* BB536, and the wild strain *Lactobacillus paracasei* N 24 after incubation with PE at different concentrations.

		Log_10_
*L. rhamnosus* GG	Baseline	8.83 ± 0.09^a^
ATCC 53103	PE (0.085 mg/ml)	9.20 ± 0.10^b^
	PE (0.042 mg/ml)	9.24 ± 0.07^b^
	PE (0.028 mg/ml)	9.26 ± 0.04^b^
*Lactobacillus paracasei*	Baseline	8.65 ± 0.15^a^
N24	PE (0.085 mg/ml)	8.70 ± 0.08^a^
	PE (0.042 mg/ml)	8.79 ± 0.10^a^
	PE (0.028 mg/ml)	8.74 ± 0.09^a^
*Bifidobacterium animalis*	Baseline	9.15 ± 0.05^a^
BB12	PE (0.085 mg/ml)	9.15 ± 0.12^a^
	PE (0.042 mg/ml)	9.44 ± 0.10^b^
	PE (0.028 mg/ml)	9.55 ± 0.07^b^
*B. longum*	Baseline	9.77 ± 0.03^a^
BB536	PE (0.085 mg/ml)	9.54 ± 0.10^a^
	PE (0.042 mg/ml)	9.57 ± 0.12^a^
	PE (0.028 mg/ml)	9.44 ± 0.16^a^


### Effect of PE on Lipid Content

**FIGURE 5:**
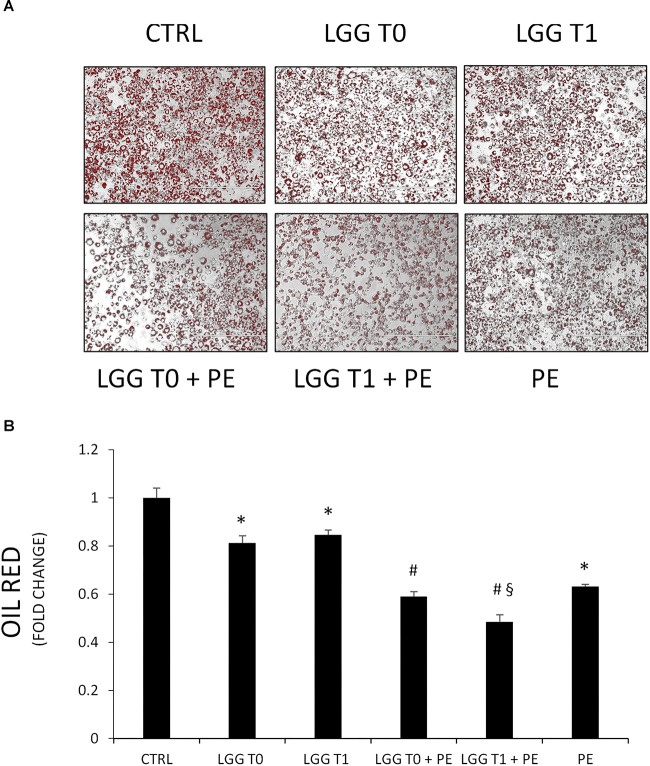
**(A)** Representative Oil red O staining of 3T3-L1 cells in absence and in presence of PE, LGG T0, LGG T1, LGG T0 + PE, and LGG T1 + PE. **(B)** Lipid content was quantified with Oil Red O staining (mean ±*SD*, ^∗^*p* < 0.05 versus control; ^#^*p* < 0.05 versus LGG T0 and LGG T1; ^§^ *p* < 0.05 versus LGG T0+PE).

Pomegranate extract- and LGG-filtered SB significantly decreased the triglyceride content compared with the control group (Figure 5A,B). However, the effect of filtered SB derived from cells incubated with PE (LGG-T1) or without (LGG-T0) was similar. The simultaneous treatment of 3T3-L1 murine pre-adipocytes with PE- and LGG-filtered SB significantly decreased the triglyceride content compared with the treatment of LGG-filtered SB alone. Moreover, these data evidenced that combination treatment of PE and LGG-T1 was the most effective in reducing triglyceride content and intracellular lipid accumulation.

The treatment of 3T3-L1 murine pre-adipocytes with LGG cellular extract (CE), derived from cells incubated with PE (CE+PE) or without (CE), had no effect on intracellular lipid accumulation compared with the control group (Control: ABS 490 nm = 0.200 ± 0.07; CE: ABS 490 nm = 0.187 ± 0.09; CE+PE = ABS 490 nm = 0.190 ± 0.05).

### Effect of PE on Adipogenic Markers

**FIGURE 6:**
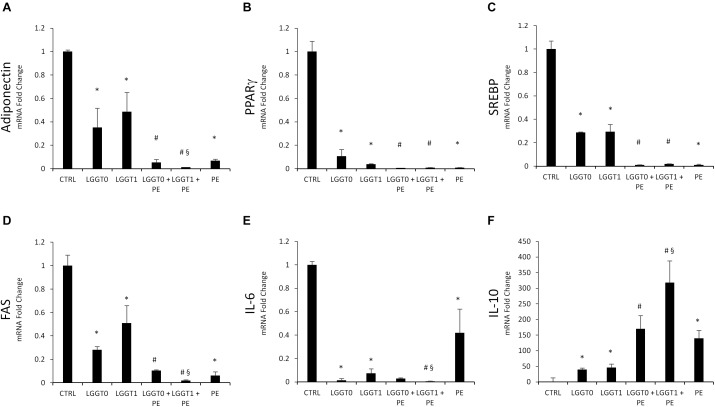
**(A–F)** Analysis of gene expression by qRT-PCR of lipogenic pathway in 3T3-L1 cells in absence and in presence of PE, LGG T0, LGG T1, LGG T0+PE, and LGG T1+PE. Results are expressed as the means ± SD of four experiments performed in triplicate (^∗^*p* < 0.05 versus control; ^#^*p* < 0.05 versus LGG T0 and LGG T1; ^§^ *p* < 0.05 versus LGG T0+PE).

The mRNA expression levels of the main transcriptional factors involved in adipocyte differentiation were significantly less expressed in 3T3-L1 cells treated with PE- and LGG-filtered SB. Particularly PE, LGG-T0, and LGG-T1 were able to decrease gene levels of Adiponectin, PPAR-γ, SREBP, FAS, and IL-6 and to increase gene levels of IL-10 (Figure 6A–F).

The simultaneous treatment of 3T3-L1 murine pre-adipocytes with PE- and LGG-filtered SB (LGGT0 and LGGT1) significantly decreased mRNA expression levels of the main transcriptional factors involved in adipogenesis, compared with the treatment of LGG-filtered SB (LGGT0 and LGGT1) alone (Figure 6A–E).

The simultaneous treatment of 3T3-L1 murine pre-adipocytes with PE- and LGG-filtered SB (LGGT0 and LGGT1) significantly increased mRNA expression levels of IL10, compared with the treatment of LGG-filtered SB (LGGT0 and LGGT1) alone (Figure 6F). Moreover, these data evidenced that combination treatment of PE+LGG T1 was the most effective in reducing mRNA expression levels of Adiponectin, IL-6, FAS, and in upregulating IL-10 (Figure 6A,D–F).

## Discussion

The beneficial effects of pomegranate fruit and/or juice consumption have received considerable scientific interest (Basu and Penugonda, 2009). Many investigators have reported that PEs, made from a waste product of the processing factories, have a free radical scavenging and potent antioxidant capacity (Panichayupakaranant et al., 2010; Fischer et al., 2011). PE used in the present study was soluble in water, and characterized through HPLC–PDA–ESI/MS*^n^* for its phenolic and anthocyanin content. In agreement with previous reports (Fischer et al., 2011; Qu et al., 2012; Romeo et al., 2015), ellagitannins are the predominant class of phenolic compounds in pomegranate peel and marc (a by-product made up of seeds and peels), since they represent over the 99% of the total content of pomegranate phenolics. The major ellagitannins of pomegranate by-products, as well as pomegranate products (fruit and juice), are punicalagins (Gil et al., 2000; Fischer et al., 2011; Qu et al., 2012).

Pomegranate extract contains high percentages of phenolic compounds and showed antioxidant activities in a concentration-dependent manner as shown for both the DPPH and superoxide anion scavenging assay.

It has been reported that dietary plant polyphenols are able to selectively modulate the growth of susceptible microorganisms (Tabasco et al., 2011). Plant extracts commonly inhibit bacterial growth, but the magnitude of the effect depends on the composition of the extract and the type of bacterial strain.

Results obtained in our experimental conditions demonstrated that high concentrations of pomegranate polyphenols exert antimicrobial activity on some pathogen strains such as *L. innocua* and *S. aureus.* These results are in agreement with studies of Panichayupakaranant et al. (2010), Fawole et al. (2012), and Su et al. (2012).

However, at concentrations of PE lower to 0.34 mg/ml, although none inhibitory activity concentrations was detected against the probiotic strains, only a slight increase in growth of LGG was evaluated. These results are not in agreement with studies of Li et al. (2015) and of Neyrinck et al. (2013) that demonstrated that pomegranate polyphenols may potentially work as prebiotics.

Obesity is a condition in which the lipids have accumulated leading to expansion of the adipose tissue that acts as a metabolic and endocrine organ. The molecular mechanisms that modulate pre-adipocytes growth, differentiation, and lipogenesis of fat cells have been subjected to extensive studies (Vanella et al., 2012; Stechschulte et al., 2014; Moseti et al., 2016; Palmeri et al., 2016; Waldman et al., 2016; Carpene et al., 2018).

During adipocyte differentiation, preadipocytes differentiate into mature adipocytes (Lefterova and Lazar, 2009). Increased fat accumulation is strongly correlated with cell number and/or size of adipocytes (Jiang et al., 2008).

It has been reported that the degree of obesity is related to the differentiation of preadipocytes in adipocytes and with enlarged adipocytes in adipose tissues (Wang and Jones, 2004).

Other authors reported that PEs were able to suppress preadipocyte differentiation and adipogenesis and to ameliorate fatty liver in the rats with obesity and type 2 diabetes (Xu et al., 2009).

In agreement with data of Moon et al. (2012) and Park et al. (2014) in our experimental conditions PE, LGG-T0, and LGG-T1 resulted in a significant reduction in lipid accumulation in 3T3-L1 cells during differentiation into adipocytes suggesting that PE, LGG-T0, and LGG-T1 are able to suppress adipocyte differentiation. However, the treatment with LGG-filtered SB derived from cells incubated with PE (LGG-T1) or without (LGG-T1) was similar. In our experimental conditions, in fact, it was not observed the prebiotic effect demonstrated by other authors. Moreover, the combination treatment of PE and LGG-T1 was the most effective in reducing intracellular lipid accumulation. These data demonstrate that, even if in our experimental conditions it was not observed a prebiotic effect, filtered SB obtained from LGG incubated with PE (LGG-T1), might contain, besides the beneficial bacterial secreted bioactive compounds, also small amounts of PE-derived bioactive compounds. The latter, present in small amounts in LGG-T1, would not be able to exert higher beneficial effects than LGG-T0, but in combination with PE could have potential synergistic health benefits.

Adipocyte-specific peroxisome proliferator-activated receptor-γ (PPARγ) is involved in the early stage of adipocyte differentiation (Rosen et al., 2000) regulating the expression of adipogenic genes such as fatty acid synthase (FAS) and sterol regulatory element-binding proteins (SREBP) and then triggering the accumulation of fat in the cells (Kawada et al., 2001; Berger and Moller, 2002).

Adipose tissue is not only a primary fat reservoir, but it is also an endocrine organ which controls lipid homeostasis. Altered levels of adipose tissue-derived adipokines can contribute in developing of inflammation, resulting in impaired lipid metabolism (Armani et al., 2010). In chronic inflammation, proinflammatory cytokines such as IL-6 are upregulated while antiflammatory cytokines such as IL-10 are downregulated (Kershaw and Flier, 2004; Bradley et al., 2008; Lira et al., 2012; Liu et al., 2018).

Our results demonstrate that the combination treatment with PE+LGG-T1 significantly downregulated the mRNA levels of adiponectin, FAS, and IL-6 and upregulated IL-10.

We can conclude that the combination treatment with PE+LGG-T1 possesses anti-inflammatory properties and it is able to inhibit the adipocyte differentiation by modulating the expression levels of key adipogenic transcription factors involved in adipogenesis.

## Conclusion

Pomegranate extract- and LGG-filtered SB significantly decreased intracellular lipid accumulation. A synergistic effect of probiotics and polyphenols contained in PE was observed. Moreover, our results evidenced that combination treatment of PE+LGG T1 was the most effective in reducing mRNA expression levels of Adiponectin, IL-6, FAS, and in upregulating IL-10.

These results evidenced that probiotics and polyphenols contained in PE may affect adipogenesis *in vitro.* Moreover, our results demonstrate that the synergistic properties of combining foods such as pomegranate and probiotics may exert combined health benefits.

Then pomegranate and probiotics such as LGG strain may contribute in development of new nutraceutical/probiotic-based remedies to prevent and to treat obesity.

## Author Contributions

VS, LV, CR, CC, FR, and GB collected research articles, conceived the experiments, analyzed the results, and wrote the manuscript. CR, MR, SF, and NT conducted the experiments.

## Conflict of Interest Statement

The authors declare that the research was conducted in the absence of any commercial or financial relationships that could be construed as a potential conflict of interest.
